# Supplementary material for: Efficacy of targeted indoor residual spraying with the pyrrole insecticide chlorfenapyr against pyrethroid-resistant Aedes aegypti
Source: PLoS Negl Trop Dis. 2021 Oct 4;15(10):e0009822. doi: 10.1371/journal.pntd.0009822 (PMC8516273; doi:10.1371/journal.pntd.0009822)
Supplement: S1 Table — (DOCX) [file pntd.0009822.s001.docx]

**S1 Table. Results from a Generalized Linear Mixed Model (GLMM) quantifying the significance in delayed mortality between control and treatment measures (control used as baseline).**

|  |  | GLMM | |
| --- | --- | --- | --- |
| Days post TIRS | Treatments | Coefficient (std. error) | P-value |
| 1 | Chlorfenapyr | 0.998 (0.05) | **0.0001** |
|  | Control |  |  |
| 14 | Chlorfenapyr | 0.869 (0.04) | **<0.0001** |
|  | Control |  |  |
| 30 | Chlorfenapyr | 0.990 (0.02) | **<0.0001** |
|  | Control |  |  |
| 60 | Chlorfenapyr | 0.877 (0.07) | **<0.0001** |
|  | Control |  |  |
| 150 | Chlorfenapyr | 0.834 (0.02) | **<0.0001** |
|  | Control |  |  |
| 210 | Chlorfenapyr | 0.630 (0.008) | **<0.0001** |
|  | Control |  |  |
| 240 | Chlorfenapyr | 0.549 (0.05) | **0.0008** |
|  | Control |  |  |
| 270 | Chlorfenapyr | 0.353 (0.05) | **0.0094** |
|  | Control |  |  |
| 300 | Chlorfenapyr | 0.353 (0.06) | **0.0164** |
|  | Control |  |  |
| 330 | Chlorfenapyr | 0.166 (0.01) | **0.0002** |
|  | Control |  |  |
| 360 | Chlorfenapyr | N/A | 1 |
|  | Control |  |  |
