# Supplementary material for: Efficacy of targeted indoor residual spraying with the pyrrole insecticide chlorfenapyr against pyrethroid-resistant Aedes aegypti
Source: PLoS Negl Trop Dis. 2021 Oct 4;15(10):e0009822. doi: 10.1371/journal.pntd.0009822 (PMC8516273; doi:10.1371/journal.pntd.0009822)
Supplement: Attachment — Submitted filename: Reviewer_comments.docx [file pntd.0009822.s002.docx]

**Reviewer's Responses to Questions**

**Reviewer #1: The objective of this study is simple, and clearly stated – to evaluate the residual efficacy of Sylando TIRS against field derived Aedes aegypti. There are just a few suggestions to improve description of the methods.**

*We thank the reviewer for the positive comments.*

**One major point in the Methods which is very unclear is the replication – how many experimental houses were used in the study? In some places it seems like one pair of houses was used, one for Sylando and one for control, and in some places it seems like multiple pairs of houses were used. I think, from Figure 2, 2 houses were used, and at each time point 3 releases were done on sequential days in these 2 houses, is that right? The figure and the text should be adapted to make this clear.**

The reviewer is correct. We used two houses and replication was based on releasing cohorts of mosquitoes on different days. We corrected the text and figures to make clear we had 2 houses but still maintained replication within the study with sequential releases.

**When describing the results in the Abstract and in the Discussion it is important to note that the mosquitoes used for the experiment were a recently colonised lab strain, derived from local collections 4 generations previously. This is important because the fact that wild mosquitoes were not used is a minor limitation of the study, particularly because the rate of conversion of chlorfenapyr is different in different populations of mosquito and may affect the results. Releases were performed over 12 months – was a new collection made and reared to F4 each time, or was it the same colony, which would therefore have been much older than F4 by the end of the experiment?**

We thank the reviewer for this observation. While we provide information about the susceptibility status, and reference papers describing the mosquito strain, we decided to expand our report of the susceptibility status of Aedes aegypti, so the readers more clearly can see that the populations are indeed resistant. Indeed, our UCBE protocols require re-seeding the populations with eggs from the field every 6 months. We now add information of the CDC bottle bioassays in the populations prior to the study as well as at the 6 month mark, to show that phenotype was maintained throughout the 12-month evaluation. Additionally, we report information on the frequency of 2 mutations from the sodium channel commonly associated with pyrethroid resistance (1010 and 1534). Our data shows that the populations were resistant throughout the study.

**It is not clear in the ‘Results’ section of the Abstract whether the reference to 48h means after 48h of exposure or scored 48h after exposure. From Figure 2 I think it’s the latter, but it is not totally clear.**

*It was 48h after exposure. Corrected.*

**Line 73 – I would describe this study as ‘semi-field’ not ‘field’.**

As field trials could be both fase II and III, we deleted “in field conditions” to be more inclusive about the need for future work on this new chemistry.

**Generally the Methods are well written and clear, and I like the map and photos in Figure 1 very much.**

Thank you.

**Lines 205-206 – ‘An evaluation for these cohorts was extended up to seven days’ - this wording is very unclear, and I think you mean ‘held mosquitoes for 7 days after recapture to score delayed mortality’?**

Noted. We modified the text as follows “Exposed mosquitoes were held for 7 days and monitored daily for signs of intoxication to quantify “delayed mortality”….”

**Table 1 – instead of the superscript footnotes, I suggest explaining these points in a more full table legend. Also, it is correct that exactly 100 mosquitoes were released in each experimental release? Even though that is what the method asked for, I would be surprised if exactly 100 were released each time – this has an impact when you use the number released to calculate mortality**.

We expanded the table legend, as suggested by the reviewer. As we indicate, we released exactly 100 female mosquitoes per replicate. The UCBE insectaries have capacity of producing thousands of mosquitoes per week, as part of their ongoing insecticide evaluation operations. We split the 100 mosquitoes in small cups with 50 of them to allow counting individuals and split releases in multiple rooms.

**Equation on line 249-250 – explain all terms**.

All missing terms (α, ε) are now mentioned in the text.

**Reviewer #1: The analysis for Figure 5 could be better described so that it can be interpreted more easily, but otherwise the results are analysed and presented well.**

We now explain interpretation of smooths in more detail: “We fitted f(Days), the non-linear term of mortality and days since TIRS, by applying a penalized cubic spline function to the data and a Gaussian link function to fit the model. The parameter f(Days) was fitted separately to the control and chlorfenapyr data. Exploration of fitted f(Days) allowed assessing the temporal trend in *Ae. aegypti* mortality after TIRS. Specifically, since f(Days) describes the non-linear fit of the time since TIRS application to the mortality data, we used the parameter’s 95% credible interval (95%CI) to quantify: 1) if the 95%CI of f(Days) differed significantly between control and chlorfenapyr treatments; and 2) at what time point the predicted non-linear fit for chlorfenapyr (with 95% CI) went from positive to negative, indicating a loss of impact of the insecticide on mosquito mortality.”

**Lines 269-270 – 3 values are given for 5 time points, but surely there should be 5?**

Apologies for missing this. We now add the values for the remaining observations.

**Lines 271-273 – this is not clearly described, which mosquitoes were used to test susceptibility, a subset of that same generation? Was this done at each time point, or just at the start of the experiment**.

We used a subset from the population that originated the experimental mosquitoes. UCBE rears thousands of mosquitoes, and we conducted the susceptibility with the siblings of the group used for releases. Also, we monitored susceptibility at the beginning and the middle of the study, when the population was mixed with larvae from field-collected eggs. While in the first draft we show the values for the starting population, we now include the values after mixing. We also added information from genotyping of mosquitoes we conduct regularly in the lab. We feel this draft now more clearly shows the ‘resistance’ status of the population.

**Line 305 – be more specific about the time when mortality was scored than ‘delayed mortality’ – was this 7 days post-exposure?**

Corrected. Yes, we measured in days post-exposure.

**Figure 5 – This is not clear to me, and it would be good to explain a bit more. When you say there was a ‘reduction in mortality’, reduction compared to what? I don’t understand where the negative values come from, or what ‘lower mortality than expected under the hypothesis of no association’ means**.

We realize that the plot, as it was, did not provide a clear link to our interpretation. To make things easier to interpret, now we modify Figure 5 to include a model for the chlorfenapyr data and another for the control data. This way, one can see how for control houses the effect of mortality does not differ from 0, whereas for chlorfenapyr we identify a rapid change in mortality consistent with loss of residual power. The methods section was modified as follows: “The parameter f(Days) was fitted separately to the control and chlorfenapyr data. Exploration of fitted f(Days) allowed assessing the temporal trend in *Ae. aegypti* mortality after TIRS. Specifically, since f(Days) describes the non-linear fit of the time since application to the mortality data, we used the parameter’s 95% credible interval to quantify: 1) if the 95%CI of f(Days) differed significantly between control and chlorfenapyr treatments; and 2) at what time point the predicted non-linear fit (with 95% CI) went from positive to negative, indicating a loss of impact of the insecticide on mosquito mortality.”

Below we provide the new figure 5 and its updated legend:

Figure 5. Generalized Additive Linear Mixed Model (GAMM) fitted to the association between mortality [s(Mortality)] and days since TIRS application [f(Days)] for the control and chlorfenapyr houses. The gray vertical line shows the threshold of change from positive to negative impact of chlorfenapyr on mosquito mortality.

**Reviewer #1: The conclusion in the first paragraph of the Discussion is very clear in scope and significance, and supported by the data from this study. Some of the other statements of Conclusion are less well supported, and sentences in the Discussion are not so clear, as described below**.

**In the last sentence of the abstract, I would like to see more explanation of how this study shows that chlorfenapyr TIRS would be ‘feasible’ in these circumstances**.

As that statement requires understanding predictions of a mathematical model that shows that increasing residual power allows for earlier intervention start, we deleted it from the abstract to focus our conclusions on the findings of the study.

**Lines 408-410 – you have not really discussed ‘large scale entomological impacts of chlorfenapyr’, what might these be, beyond delayed mortality**?

We modified that sentence, which now reads “Future studies should evaluate the entomological impact of chlorfenapyr TIRS against Ae. aegypti in field randomized trials, providing evidence of the value of this new chemistry for the management of pyrethroid resistance and the prevention of Aedes-borne viruses.”

**Lines 111-114 –The claimed link between the mode of action of chlorfenapyr and insecticide relevance mechanisms should be explained**.

We assumed the reviewer meant ‘insecticide resistance mechanisms’. We edited the sentence, as follows “The mode of action of chlorfenapyr on an insect’s metabolism is particularly relevant for the control of vectors harboring metabolic insecticide resistance mechanisms (e.g., cytochrome P450, glutathione S-transferases), as increased metabolic activity increases the activation of the toxin and increase mosquito mortality”

**Lines 399-400 – you state the efficacy can be increased by 90% when IRS lasts 5 months, but compared to what?**

We edited the sentence, which now reads “Mathematical modeling showed that effectiveness of TIRS can be increased up to 90% compared to not conducting TIRS when residual efficacy of the insecticide lasts 5 months”

**Reviewer #1: Line 96 – ‘IRS’ not ‘ISS’?**

Done.

**All statements in lines 347-364 need to be supported by references**.

Done

**Lines 369-370, for readers who do not know, please state the significance of these 4 insecticides**.

Done

**Line 378 – I think you mean ‘>80%’ not <80%’?**

Correct! Thanks for catching that typo.

**Lines 381-394 – this paragraph is not linked to the current experiment, and I don’t see the value in including this text.**

We included this paragraph to outline the complexities of Pyrrole insecticides in assessments of susceptibility and their potential for insecticide resistance management. We kept the paragraph, but after editing to give it more context.

**Reviewer #1: This manuscript very nicely presents a solid experiment which demonstrates that Sylando, an IRS formulation of chlorfenapyr, is effective for 7 months when used in a TIRS strategy in semi-field experimental conditions. The method has been published previously, but the demonstration of the improved performance of this formulation against Aedes aegypti is a valuable novel finding. The study is clearly described, with just a few points of clarification needed, and a careful edit to correct some minor English errors.**

Thank you.

**I think that TIRS should be mentioned in the title, since the mode of delivering the insecticide is part of the novelty and interest of this paper**.

Done. Title now reads: “Efficacy of targeted indoor residual spraying with the pyrrole insecticide chlorfenapyr against pyrethroid-resistant Aedes aegypti”

**Reviewer #2: Dear Authors,**

**Thank you for submitting this interesting manuscript.**

**STRENGTHS OF THE MANUSCRIPT**

**The study design is simple, results are unambiguous and clearly reported.**

**Chlorfenapyr is a new product in vector control and due to its late acting properties, it needs the assessment of delayed mortality, something that is not common in this kind of studies.**

**Sylando 240 SC shows a good potential as an IRS product to overcome insecticide resistance**

Thank you for comments.

**MAJOR COMMENTS**

**A positive control like a pyrethroid or a carbamate used in IRS would have added strength to the study.**

We have conducted a previous study in the same houses and with the same methods and strain utilizing the carbamate Bendiocarb. The discussion mentioned the comparison between our findings and the finding with bendiocarb: “Alternative chemistries (to which *Ae. aegypti* is susceptible) exist, and the carbamate bendiocarb has provided not only to control pyrethroid-resistant *Ae. aegypti* [] but also to exert mortalities >80% for up to 4 months in experimental houses []. Our study shows that, in experimental houses, chlorfenapyr can extend TIRS residual efficacy against *Ae. aegypti* up to seven months.”

As this is a major issue identified by the reviewer, we now add Figure 6 comparing the acute mortalities from bendiocarb and chlorfenapyr. We decided to compare acute mortalities because we did not measure delayed mortality for bendiocarb.

Figure 6. Comparison of acute mortality after TIRS application of bendiocarb (Dunbar et al. [7], blue line) and chlorfenapyr (orange, present study) in experimental houses from Merida, Mexico.

**MINOR COMMENTS for Authors**

**Authors describe a “replicated system of two experimental houses”, Lines 132-133, with distance between them ranging “from 0.3 to 2 km” Lines 136. I read this as 4 houses with 2 treatments and 2 controls, otherwise the distance between 2 houses would be a single value. On the other hand, from Figure 2 and Table 1 it seems the experimental houses were 2 only, according to the number of mosquitoes released per each time point (300+300). I suggest to better explain this in the text.**

As also addressed in Reviewer 1 comments, we now explain better the experimental setup and the fact that we had 2 houses (one per treatment) and that replication was done by conducting sequential releases on each house.

**In the manuscript it is not reported where mosquitoes were kept after being collected from the experimental houses. In light of the high delayed mortality recorded, this is an important information for understanding if mosquitoes had the possibility to fly and be active after chlorfenapyr exposure, continuing to convert chlorfenapyr to its metabolite.**

The reviewer is correct. We did not provide details of this component. We kept surviving mosquitoes inside a bugdorm (30x30x30 cm) inside the UCBE insectary, which has constant laboratory conditions of 26C +-1 and 75+-5%RH. This is now added to the text.

**“where furniture could not be removed (e.g., built-in kitchen or closet cabinets) it was sealed with window screening” Lines 157-158. I understand here that mosquitoes could not access the inner parts or land on furniture, but I wonder if they could still fly under or behind it, exploiting the gaps with walls and floors. Maybe it would be worth to explain this.**

This only applied to the kitchen cabinets. We placed netting on gaps to make sure that mosquitoes do not get trapped in them (a pilot study when we first worked in the experimental houses showed high mosquito loss due to their entrance inside cabinets, and high mortality because they got trapped there).

**Please, check the following sentences as it looks to me that Methods do not match with Results:**

**Lines 209-212 “On each house, we placed three unsprayed control cups (250 mL) containing 10 JP strain females each during the 24-h exposure period to have an independent measure of mosquito mortality due to the temperature and humidity conditions of the experimental houses. This measure was estimated at the +5, +7, +8-12 months post-application…”; and Lines 268-271 “Mortality within cups left inside houses to monitor natural mortality averaged 4.4 ± 1.3%, 1.5 ± 0.7% and 5.0 ± 1.7% (Mean ± SEM) for evaluations from +8, +9, +10, +11 and +12 months post-application, respectively…”**

As also mentioned in Reviewer 1 comments, we now provide details of all the rates.

**Check for typos Lines 160 and 280.**

Done.

**Line 345. It is the first time MoH appears in the manuscript. I suggest writing it in extenso.**

Done.
